# Control over self and others’ face: exploitation and exploration

**DOI:** 10.1038/s41598-024-66316-2

**Published:** 2024-07-05

**Authors:** Wen Wen, Jie Mei, Hakan Aktas, Acer Yu-Chan Chang, Yosuke Suzuishi, Shunichi Kasahara

**Affiliations:** 1 Department of Psychology, Rikkyo University, 1-2-26 Kitano, Niiza City, Saitama, 352-8558 Japan; 2 International Research Center for Neurointelligence, University of Tokyo, Tokyo, Japan; 3 Bogazici University Cognitive Learning and Robotics Lab, Istanbul, Turkey; 4 Sony Computer Science Laboratories, Inc., Tokyo, Japan; 5 Okinawa Institute of Science and Technology Graduate University, Okinawa, Japan

**Keywords:** Sense of agency, Control, Exploitation, Exploration, Sensitivity, Criterion, Human behaviour, Cognitive neuroscience

## Abstract

The face serves as a crucial cue for self-identification, while the sense of agency plays a significant role in determining our influence through actions in the environment. The current study investigates how self-identification through facial recognition may influence the perception of control via motion. We propose that self-identification might engender a belief in having control over one’s own face, leading to a more acute detection and greater emphasis on discrepancies between their actions and the sensory feedback in control judgments. We refer to the condition governed by the belief in having control as the exploitation mode. Conversely, when manipulating another individual’s face, the belief in personal control is absent. In such cases, individuals are likely to rely on the regularity between actions and sensory input for control judgments, exhibiting behaviors that are exploratory in nature to glean such information. This condition is termed the explorative mode. The study utilized a face-motion mixing paradigm, employing a deep generative model to enable participants to interact with either their own or another person’s face through facial and head movements. During the experiment, participants observed either their own face or someone else’s face (self-face vs. other-face) on the screen. The motion of the face was driven either purely by their own facial and head motion or by an average of the participant’s and the experimenter’s motion (full control vs. partial control). The results showed that participants reported a higher sense of agency over the other-face than the self-face, while their self-identification rating was significantly higher for the self-face. More importantly, controlling someone else’s face resulted in more movement diversity than controlling one’s own face. These findings support our exploration–exploitation theory: When participants had a strong belief in control triggered by the self-face, they became highly sensitive to any sensorimotor prediction errors, leading to a lower sense of agency. In contrast, when the belief of control was absent, the exploration mode triggered more explorative behaviors, allowing participants to efficiently gather information to establish a sense of agency.

## Introduction

When we observe the movements of our body, we typically have no doubt that we initiated and controlled those movements. This subjective feeling of controlling our own actions, and the external events associated with them, is termed the sense of agency^1^. In daily life, we usually have a robust and reliable sense of agency over well-established motor control. For example, when we reach for a bottle of water or use a mouse to click a button on the screen, the sense of agency over our arm and the mouse cursor is almost implicit and needs no attention unless there is an unexpected muscle twist or a disturbance on the screen. In such cases, having reliable control is the default belief, and the mismatches (i.e., prediction errors) between the predicted and the actual sensory input become salient, diminishing our sense of agency^2–5^. On the other hand, when people explore unfamiliar objects, control is not guaranteed, and gaining control affects our attention allocation and decision-making^6,7^. Both aspects, error detection under the belief of control and explorations when the belief of control is absent, are important for understanding how humans interact with the environment through their actions.

Previous developmental studies have demonstrated that infants’ behaviors, such as leg kicking, gazing, and sucking, can be rapidly reinforced by the contingency between their actions and the resultant sensory feedback^8–11^. This suggests that the foundational processes for the emergence of a sense of agency and self-identification through actions are acquired at a very early stage of human development. A broad concept of self-identification refers to the association with the self. The sense of agency can play a role in this broad self-identification by linking sensory input with one’s own actions. On the other hand, the narrower concept of self-identification refers to the specific feeling of “that is me,” which can be achieved through multiple cues such as sensory input, one’s name, voice, and appearance. The identification of self exerts a significant influence on numerous cognitive processes and behaviors. For instance, individuals respond more swiftly to their own faces and names than to those of others^12–15^. The manner in which self-recognition may influence the sense of agency and the corresponding actions remains largely unexplored. Consider the scenario where, upon viewing oneself in a mirror, there is generally no doubt that one should be able to control the reflected image, which can be described as a belief in one’s ability to exert control. Does having such a belief engender a stronger sense of agency? A recent study investigating the use of self-voice suggests this to be the case, showing that the intentional binding effect—an implicit measure of the sense of agency—was more pronounced for self-voice than for other-voice^16^. However, this phenomenon may be attributed to the relatively low actual level of control and the possibility that self-identification influenced the judgment criterion for the sense of agency in ambiguous conditions.

Self-identification may influence both the criterion and sensitivity of the sense of agency by shaping the belief in control^17^. We propose that this belief in control likely affects the sensitivity towards the processing of prediction errors or regularities^17^. Specifically, the belief of having control fine-tunes our perceptual system to detect discrepancies between our actions and sensory feedback. Conversely, the absence of such a belief shifts our perceptual focus towards identifying regularities between actions and sensory feedback^18,19^. In essence, when individuals perceive reliable control, they become acutely sensitive to any minor loss of control^19^. This heightened sensitivity benefits individuals by enabling them to adjust their behavior to regain control during exploitative actions. On the other hand, in the absence of a belief in reliable control, such as during initial interactions with a new robot, the absence of clear predictions makes regularities between one’s actions and the corresponding sensory feedback valuable cues for detecting control^20,21^. It is important to note that regularities are not merely the opposite of prediction errors, as the former do not always necessitate a prediction.

**Figure 1:**
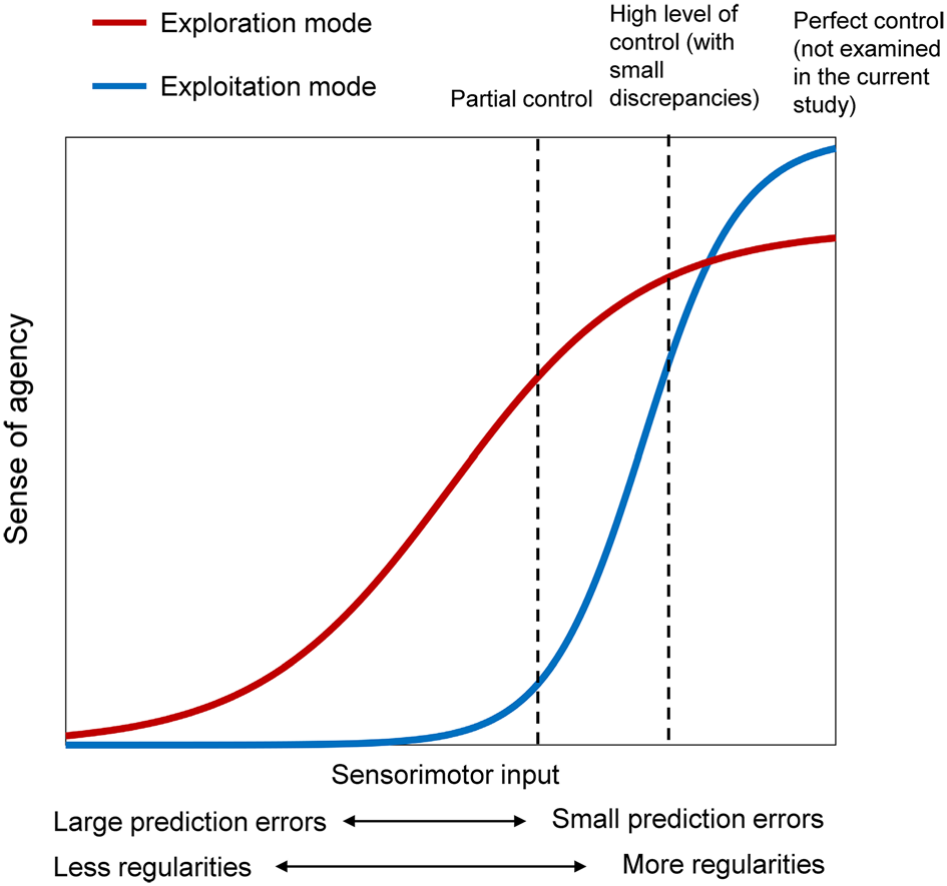
The relationship between sense of agency and sensorimotor input in the exploration (red line) and exploration (blue line) modes. In the exploitation mode, even small prediction errors can lead to a significant drop in the sense of agency, as indicated by the steep curve. This contrasts with the exploration mode, where the sense of agency increases more gradually with sensorimotor input, and the slope of the curve is gentler, indicating a more tolerant response to prediction errors.

The current study examines the impact of self-identification on the sense of agency. Self-identification can affect both the criterion and sensitivity of the sense of agency by shaping the belief in control^17^. The belief in control may amplify the sense of agency in ambiguous control situations by influencing the criterion for the sense of agency^16^. However, the effect of the belief in control on sensitivity has yet to be elucidated. We propose that a belief in control, fostered by self-identification, likely enhances the detection of prediction errors during exploitative actions. This diminishes the sense of agency more significantly in the presence of minor discrepancies between actions and sensory feedback than in the absence of control belief. In contrast, the absence of a belief in control is likely to activate the exploration mode, improving the detection of regularities between one’s actions and sensory feedback. Moreover, the action policies underpinning the sense of agency may vary between these modes^22^. To identify regularities, exploratory actions are more probable, characterized by larger and more varied movements compared to exploitative actions. This exploration–exploitation hypothesis is illustrated in Fig. 1. The exploitation model can be activated by self-identification and is associated with a sharp curve of the sense of agency depending on sensorimotor input. In this case, a slight lack of control can result in a significant drop in the sense of agency. On the other hand, the sense of agency in the exploration model is associated with a gentler curve of sensorimotor input. In addition, when the prediction error is minimum, the exploitation mode is likely to ensure a higher level of confidence in control than the exploration mode. Lastly, these two modes can switch between each other depending on the update of the belief in control.

The present study utilized a face-motion mixing paradigm powered by the state-of-the-art deep generative model, Latent Image Animator (LIA)^23^ to examine how self-identification may affect the sense of agency and actions underlying it. Faces are powerful cues for self-identification in adults^24^ and can significantly influence our beliefs about control. Recognizing one’s own face typically induces a strong belief in having control and leads to the exploitation of control, as this is consistent with our everyday experiences. In contrast, controlling someone else’s face represents a novel experience for individuals and is likely to engage the exploration mode. The former is associated with more precise, less varied movements and strict self-attributions, while the latter correlates with broader, more diverse movements and more lenient self-attributions. In summary, the experiment used a 2 × 2 (face × control) within-participant design. First, the factor of face refers to the facial structure information used to generate the face video. It was either participants’ faces (i.e., self-face) or the female experimenter’s face (i.e., other-face). The facial videos shown to participants were created by combining real-time motion data extracted from webcam footages with a static facial photograph taken prior to the experiment. Consequently, the algorithm guarantees a consistent level of control over the motion of a face, independent of the face’s appearance. Second, the factor of control refers to the extent of how well participants can control the face video. It contains two conditions, full control and partial control. In the partial control condition, the motion of the face video was driven by the mixture of participants’ motion and the experimenters’ motion. The agency rating (whether the presented face was controlled by oneself), self-identification rating (whether the presented face resembled their own), participants and the experimenter’s motions were recorded from each trial. In addition, the agency rating and self-identification rating served as both manipulation checks and key dependent variables to test the hypothesis. It is important to note that there were small spatial and temporal discrepancies between the presented face and participants’ own face even in the full control condition due to technical limitations (see Methods). Therefore, we were only able to examine the sense of agency at the level of actual control marked with broken lines in Fig. 1. The exploration–exploitation hypothesis predicts a lower sense of agency for the self-face than the other-face when there are small temporal and spatial discrepancies between one’s own movements and the presented face. This is because people are probably more sensitive to prediction errors when controlling their own face, compared to when controlling someone else’s face, resulting in a weakened sense of agency. In other words, people are more likely to notice the small lack of control when controlling their own face. In addition, this prediction only holds when the actual control is not at the highest level. If people have perfect control, their confidence of control over the self-face is likely to be higher than that over the other-face. Furthermore, the hypothesis also predicts more frequent and diverse movements for the other-face than the self-face, as the other-face triggers the exploration mode, which is associated with explorative behaviors.

## Results

### Subjective rating

**Figure 2:**
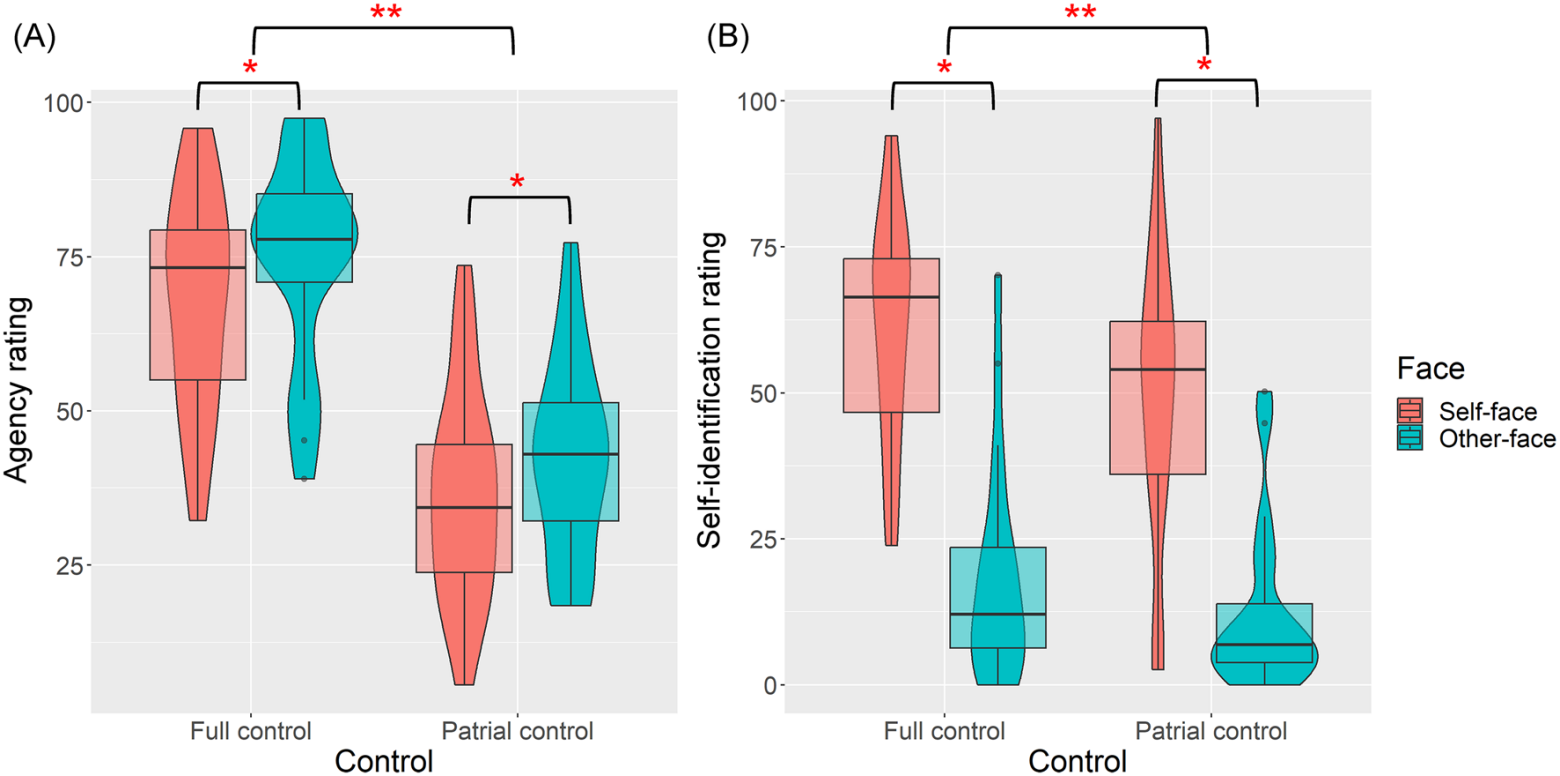
The plot of participants agency rating (**A**) and self-identification rating (**B**) over the displayed face in each condition. The violin shapes show a kernel density estimate of the data. The box plot visualizes the central tendency and dispersion of the data. The central box represents the interquartile range (IQR, the middle 50% of the data). The horizontal line inside the box marks the median. The whiskers represent the smallest and largest values within 1.5 times the IQR from the lower and upper quartiles. Points outside of the whiskers indicate outliers. The same applies for the other violin plots and box plots in Figs. 3 and 4. Note: *p < 0.05, **p < 0.01.

Figure 2A and B show the average agency rating (“How much control did you feel over the presented face?”, ranging from 0 to 100) and the self-identification rating (“How much did you feel that the presented face resembled your own?”, ranging from 0 to 100) of each participant in each trial. A 2 × 2 repeated measures ANOVA revealed a significant effect of face (*F*(1, 19) = 11.419, *p* = 0.003, partial η^2^ = 0.375) and a significant main effect of control (*F*(1, 19) = 74.310, *p* < 0.001, partial η^2^ = 0.796). The interaction between face and control was nonsignificant (*F*(1, 19) = 0.052, *p* = 0.822, partial η^2^ = 0.003). People reported a stronger sense of agency over someone else’s face than over their own face. This finding corroborates our hypothesis that self-identification diminishes, rather than enhances, the sense of agency in the presence of minor discrepancies between one’s actions and the sensory feedback. The agency rating was also significantly higher in the full control condition compared to the partial control condition. Regarding the self-identification rating, both the main effect of face (*F*(1, 19) = 40.813, *p* < 0.001, partial η^2^ = 0.682) and the main effect of control (*F*(1, 19) = 14.761, *p* = 0.001, partial η^2^ = 0.437) were significant. The interaction between face and control was nonsignificant (*F*(1, 19) = 1.683, *p* = 0.210, partial η^2^ = 0.081). People reported a stronger sense of self over their own face than over someone else’s face. Additionally, the self-identification rating was higher when they had greater control over the face. This indicates that participants considered both the visual features and the movement of the face for self-identification. Additionally, the discrepancy between the participants’ actual facial movements and the movements displayed was quantified to objectively assess the real level of control (see S1 Fig). The results confirmed that motion errors were significantly larger in conditions of partial control compared to full control. The motion errors were also slightly greater for the other-face than for the self-face. This difference is likely attributable to variations in extracting FaceMesh Keypoints from different faces (refer to Data Analyses). Nevertheless, the heightened sense of agency reported for the other-face was not due to superior actual control over the other-face but was likely the result of internal processing and the weighting of sensorimotor cues in determining the sense of agency.

### Head and facial muscle movements

**Figure 3:**
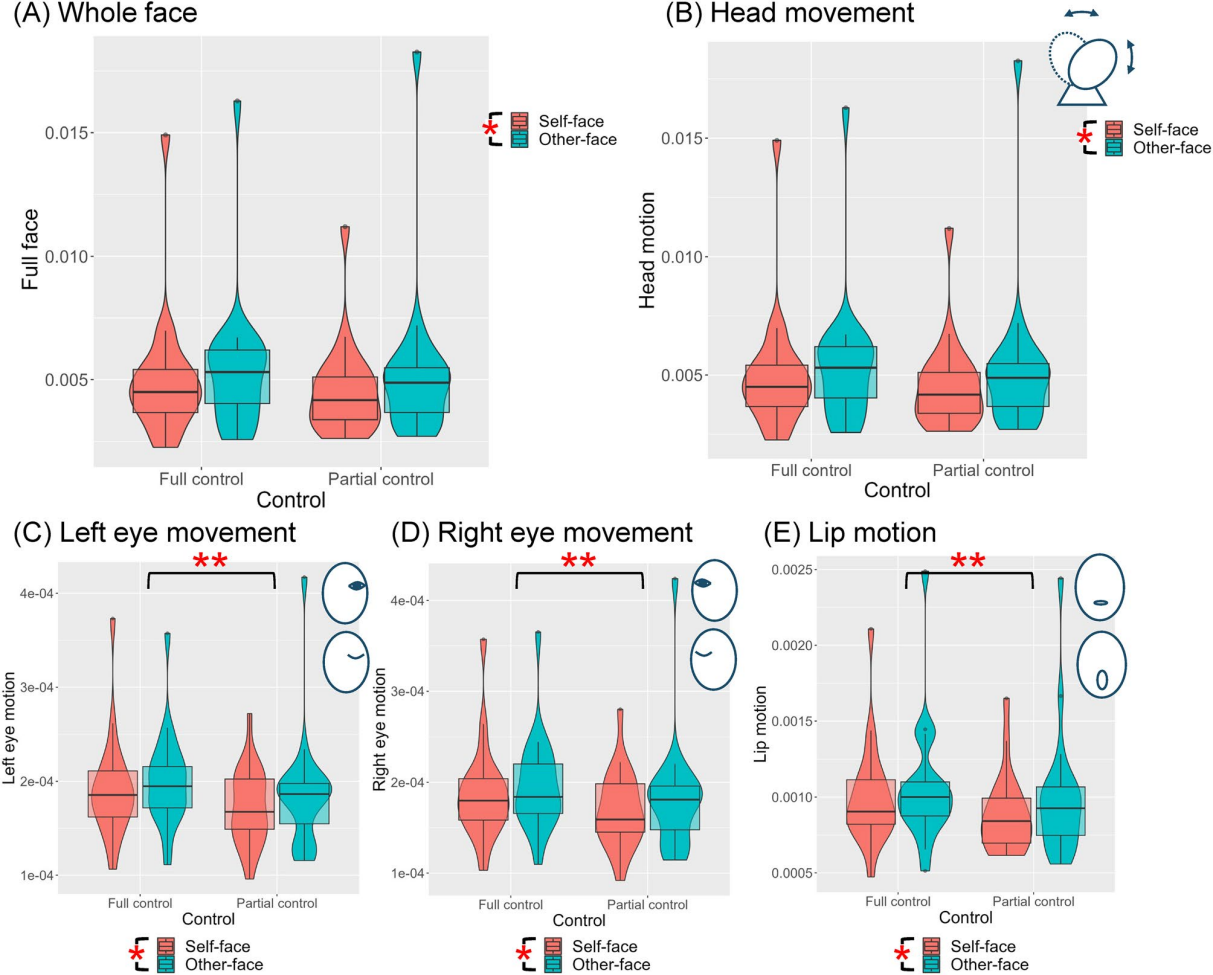
Moving distance in each condition of the whole face (**A**), the head movement (**B**), the left eye movement (**C**), the right eye movement (**D**), and the lip movement (**E**). The moving distance was significantly larger for the other-face than for the self-face. The moving distance of eyes and lips was significantly larger in the full-control condition than the partial control condition. The value of the coordinates is contingent upon the resolution of the video and, therefore, does not contain actual metric information; hence, the unit of the y-axis was omitted in the figures. Note: *p < 0.05, **p < 0.01.

The purpose of the action analysis was to discern the differences in exploitation and exploration behaviors under the two action modes, as hypothesized. To this end, we calculated two types of indices: moving distance and motion diversity. Figure 3 presents the overall moving distance for the whole face and the moving distances for each category, including head motion, left eye movement, right eye movement, and lip movement. The statistics from the 2 × 2 (face × control) repeated-measures ANOVAs are summarized in Table 1. Overall, the moving distance was significantly larger when participants controlled the other-face as compared to controlling their self-face in all regions. Moreover, there was a significant reduction in facial muscle movements, specifically in the eyes and lips, in the partial control condition compared to the full control condition.

**Table 1:** Statistics of the 2 × 2 (face × control) repeated-measures ANOVAs for each type of movement.

	Main effect of face	Main effect of control	Interaction between face and control
Whole face	*F(1, 19) = 5.373, *p* = 0.032, partial η^2^ = 0.220	F(1, 19) = 3.557, *p* = 0.075, partial η^2^ = 0.158	F(1, 19) = 0.842, *p* = 0.370, partial η^2^ = 0.042
Head	*F(1, 19) = 4.725, *p* = 0.043, partial η^2^ = 0.199	F(1, 19) = 3.099, *p* = 0.094, partial η^2^ = 0.140	F(1, 19) = 0.792, *p* = 0.388, partial η^2^ = 0.040
Left eye	*F(1, 19) = 6.696, *p* = 0.018, partial η^2^ = 0.261	**F(1, 19) = 10.725, *p* = 0.004, partial η^2^ = 0.361	F(1, 19) = 0.421, *p* = 0.524, partial η^2^ = 0.022
Right eye	*F(1, 19) = 5.688, *p* = 0.028, partial η^2^ = 0.230	**F(1, 19) = 10.853, *p* = 0.004, partial η^2^ = 0.364	F(1, 19) = 0.475, *p* = 0.499, partial η^2^ = 0.024
Lip	*F(1, 19) = 7.033, *p* = 0.016, partial η^2^ = 0.270	**F(1, 19) = 9.334, *p* = 0.007, partial η^2^ = 0.329	F(1, 19) = 1.717, *p* = 0.206, partial η^2^ = 0.083

*p < 0.05, **p < 0.01.

**Figure 4:**
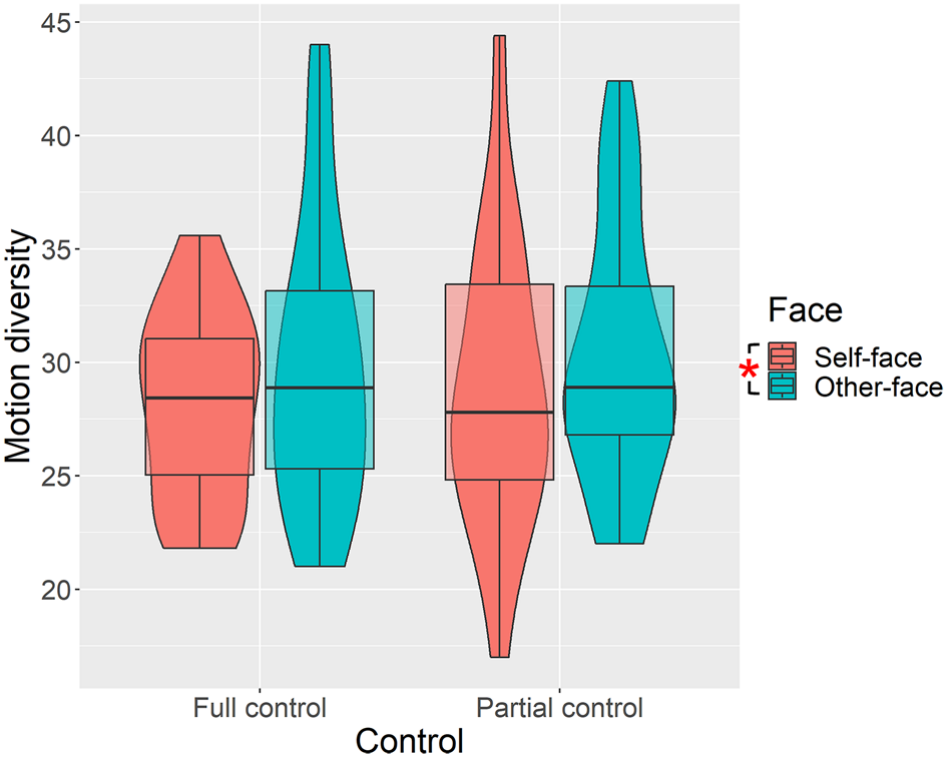
Motion diversity index in each condition. The motion diversity was significantly higher in the other-face condition compared to the self-face condition. Note: *p < 0.05.

Figure 4 further shows the index of motion diversity in each condition. The repeated-measures ANOVA revealed a significant main effect of face (*F*(1, 19) = 5.555, *p* = 0.029, partial η^2^ = 0.226). The main effect of control and the interaction between face and control was nonsignificant (*F*(1, 19) = 0.798, *p* = 0.383, partial η^2^ = 0.040; *F*(1, 19) = 0.002, *p* = 0.963, partial η^2^ = 0.000). The motion diversity was higher when controlling the other-face compared to when controlling the self-face.

## Discussion

The current study investigates how self-identification via face appearance influences the sense of agency over the face and the underlying behaviors. We propose that self-recognition is associated with a belief in control, activating the exploitation mode of the sense of agency, leading to actions that exploit control and attune the perceptual system towards detecting prediction errors. In contrast, in the absence of control belief (i.e., when controlling another person’s face), individuals exhibit exploratory behaviors, with the perceptual system adjusted to identify regularities between one’s action and sensory feedback. In short, self-identification likely leads to a more significant decrease in the sense of agency when minor discrepancies in sensorimotor input are present, compared to conditions involving stimuli relevant to others. Our findings, derived from both subjective ratings and action analyses in tasks where participants controlled either their own face or someone else’s, support our hypotheses and predictions. The sense of agency was significantly lower when participants controlled their own face compared to someone else’s. This suggests that discrepancies were more significantly weighted for the self-face than for the other-face, as predicted by the exploitation mode. Crucially, our action analyses indicated that participants engaged in a wider and more diverse range of head and facial muscle movements when controlling someone else’s face, highlighting the exploratory nature of these behaviors in the exploration mode. Additionally, the results of the self-identification ratings indicated that while the synchronization of motion played a role in self-identification, the appearance of the face was predominant. This predominance facilitated a rapid establishment of the belief in control. In other words, the sense of agency may facilitate broader self-identification (i.e., associating external events with the self), but narrower self-identification is primarily determined by the appearance of the face.

In our study, errors related to movement were slightly smaller for the self-face (S1 Fig), yet the agency rating was significantly lower for the self-face compared to the other-face. This discrepancy could be due to errors being more readily detected and given greater weight in agency ratings for the self-face. Alternatively, the regularities between one’s actions and the displayed face might be more strongly weighted for the other-face. Either explanation indicates that perceptual sensitivities for errors and regularities differ substantially between the self-face and the other-face. Regularities are not simply the inverse of prediction errors, as the mechanisms underlying the processing of these cues differ^20,21^. Furthermore, it is also possible that individuals are more adept at identifying discrepancies with their own face, which could result in a diminished sense of agency for the self-face compared to the other-face^12,13,25^. Nevertheless, the findings showed that the prior belief activated by self-identification does not lead to an enhanced sense of agency as previously reported^16^. Instead, individuals are likely highly sensitive to prediction errors under the belief of control, rapidly losing the sense of agency when minor discrepancies in sensorimotor input are detected. The findings suggest a potential reduction in the sense of agency when the feature of subjective control is too closely aligned with the self, such as when designing a control interface for controlling a humanoid robot or a virtual avatar. This is akin to the phenomenon of the “uncanny valley,” where human-like figures (e.g., robots, computer-generated characters) that closely resemble humans can evoke feelings of unease or discomfort among human observers^26,27^. Research in human–machine interaction must carefully consider the impact of self-identification on control behaviors and the sense of agency.

Furthermore, action policies underlying the sense of agency remain largely unexplored. Previous studies have primarily focused on the perceptual and judgment aspects of agency while strictly limiting action freedoms, although voluntary actions determine the sensory inputs one receives and are essential for the sense of agency. Several studies have reported that the sense of agency enhances the response speed of key presses as an internal reward^7,28,29^, indicating that the sense of agency not only arises from control but also connects subjective experiences with behaviors. In our study, where the belief in control was manipulated by the type of face while actual control was evenly distributed between face types, we observed significantly larger movements and more diverse moving strategies for the other-face than for the self-face, highlighting the differences between exploratory and exploitative action policies.

There are some limitations of this study that are worth to note. The present study used self- and other-faces to manipulate the belief of control. However, due to our novel design, the face identity itself could also affect curiosity or engagement with the technology, bringing unexpected influence on both the ratings and action policies. To address this concern, we conducted additional analyses on the effect of repetition on both agency rating and moving distance since repeated exposure to the same stimuli could decrease the level of curiosity or engagement^30,31^. The result of the additional analysis showed no significant effect of repetition, suggesting that curiosity or engagement should not be the main factors driving control actions and the sense of agency. However, it is difficult to completely exclude the influence of curiosity since the experiment only had five repeats for each condition. We believe that curiosity is an important motivation of explorative behaviors when the belief of control is absent. The explorative behaviors gathers novel evidence of control, updating the prior belief of control, as suggested by the active causal inference theory^22^. Furthermore, the familiarity with one’s own face can be another reason why the sense of agency was lower compared to the other-face^32,33^. Specifically, people are very likely to detect small spatial and temporal discrepancies between their own facial and head movements and the presented face. This leads to larger prediction errors, which diminish the sense of agency to a greater extent. It is difficult to distinguish whether the lower sense of agency for the self-face was due to larger weightings on prediction errors or, alternatively, better detection of prediction errors. This is indeed a limitation of the current study due to the unique stimuli of faces. The present study did not consider the possible influence of gender because we focused on the general mechanism of exploration and exploitation modes in the sense of agency. The other-face was always a female face. Both the gender of the participants and the gender of the presented face may affect the processing sensitivity of prediction errors and self-identification. Additionally, the present study relied solely on subjective ratings to measure the sense of agency instead of employing implicit measures such as intentional bindings and sensory attenuation. This approach was intended to promote natural interaction with the stimuli, as adding implicit measures might disrupt how participants engage with the task. However, we acknowledge that relying solely on subjective ratings is a limitation of our study, as these ratings can be influenced by both the sensitivity and criterion of sense of agency^17^. To enhance both ecological and experimental validity, future studies should consider incorporating a balanced mix of subjective and implicit measures. Lastly, the present study is only an initial examination of the proposed exploration–exploitation theory. Further studies should be conducted using different methods to shape prior beliefs regarding control and to examine the sense of agency in the whole range control under various beliefs.

In conclusion, our results demonstrate that self-identification significantly influences the sense of agency and the behaviors underpinning it. Self-identification is linked with the exploitation mode, leading to heightened sensitivities to minor discrepancies in control. Conversely, interaction with non-self-relevant stimuli is associated with the exploration mode, characterized by more diverse behaviors and greater emphasis on regularities in the judgment of agency. Our exploration–exploitation theory, which suggests that the belief in control affects action policies and perceptual sensitivities towards errors and regularities, offers a valuable framework for understanding how individuals interact with their environment through their actions. Furthermore, in the future development of cybernetic societies, people may encounter more opportunities to interact with avatars or control robots using human–machine interfaces. Our findings and theory may be useful in predicting the human sense of agency and explorative behaviors in novel or familiar environments^34^.

## Methods

### Participants

Twenty-one university students (14 females and 7 males, mean age = 20.0 years, *SD* = 1.3) were recruited from a campus-based participant database for the experiment. All participants had normal or corrected-to-normal visual acuity and typical motor abilities. The experiment was conducted according to the principles of the Helsinki Declaration and was approved by the ethics committee of the Department of Psychology at Rikkyo University (ref: 23–08). Written informed consent, including consent to the use and recording of their facial video, was obtained from all participants prior to their participation. Participants’ recorded facial videos were only used for analysis purposes and were not made public within the dataset. They received compensation for their participation. One participant was excluded from the analysis due to a misunderstanding of the instructions.

Our hypothesis centers on the distinction between the two modes of agency. As such, we calculated the d’ of the signal detection theory^35^ for detecting both an increase and a decrease in control from the raw data (10.17632/bbc5ynn2bm.3) of a previously published study^36^ and used this for the power calculation. The original dataset from the prior study encompassed 54 participants, and the effect size (Cohen’s d) was 0.617 for the difference in d’ between increasing and decreasing control detection. This suggests that a sample size of 18 would be sufficient to achieve a power of 0.8 (using a one-tailed independent t-test, α = 0.05) to discern the difference between the two modes.

### Experimental task and procedure

The experimental task was programmed using TouchDesigner (Derivative). It incorporated the open-sourced LIA (Latent Image Animator; https://github.com/wyhsirius/LIA)^23^ and ran on a high-performance PC. During the experiment, participants’ face video was captured in real-time using two webcams (Brio ultra HD pro, Logicool) mounted on two 24-inch LED monitors (FlexScan EV2456, EIZO). One webcam and monitor set was used to capture participants’ faces and to display the experimental stimuli and instructions, while the other set was used by the experimenter. The two monitors were positioned on separate tables with partitions in between, ensuring that participants and the experimenter could not see each other during the experiment. The average latency between a participant’s motion and the displayed video was approximately 300 ms.

**Figure 5:**
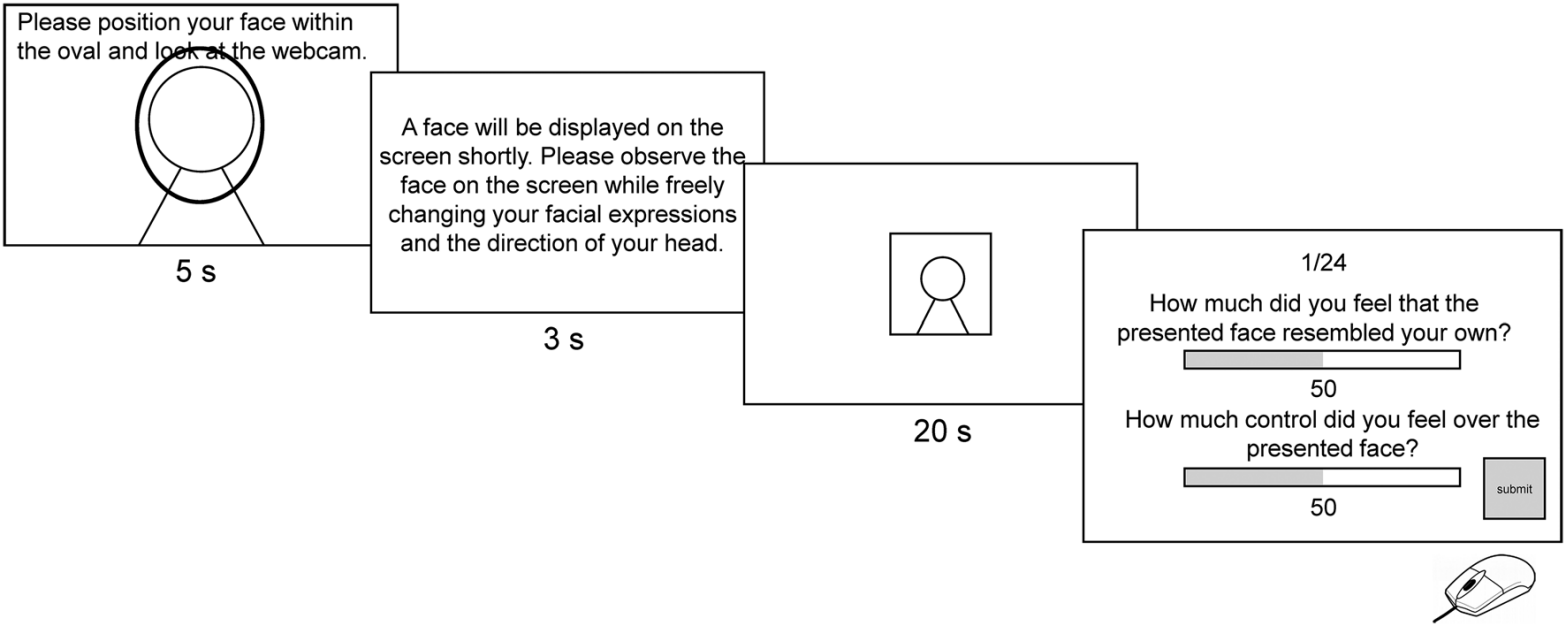
Timeline of the experimental task.

Figure 5 illustrates the timeline of a trial. At the onset, an oval displaying participants’ real-time face video was shown for 5 s. Participants were instructed to look directly at the webcam for camera calibration during this phase. Subsequently, the face video and oval were replaced with an on-screen instruction, informing participants that a face would appear, and they were encouraged to move their head and alter their facial expressions freely to gauge their control over the displayed face. Participants were informed that the face might either be theirs or the experimenter’s. Additionally, they were advised against remaining static upon viewing the face stimulus. Upon any motion from participants, the face stimulus would move accordingly. This instruction remained on screen for 3 s, after which it was substituted with a 256 × 256 px face video which is displayed as upscaled 384 × 384 px in a 1920 × 1080 px monitor. The movement of the face video was contingent on the motion of participants and/or the experimenter, depending on the control condition (full control or partial control, see below for the design of the experiment). After a 20-s period of exploration, participants answered two rating questions by adjusting a bar on the screen beneath each query using the mouse. The first question was, “How much did you feel that the presented face resembled your own?” (i.e., the self-identification rating). The second inquired, “How much control did you feel over the presented face?” (i.e., the agency rating). Their ratings (ranging from 0 to 100) were displayed beneath each respective bar, and they clicked a button to finalize their responses. Participants were guided to rely on intuition for these ratings and to use their own judgment.

**Figure 6:**
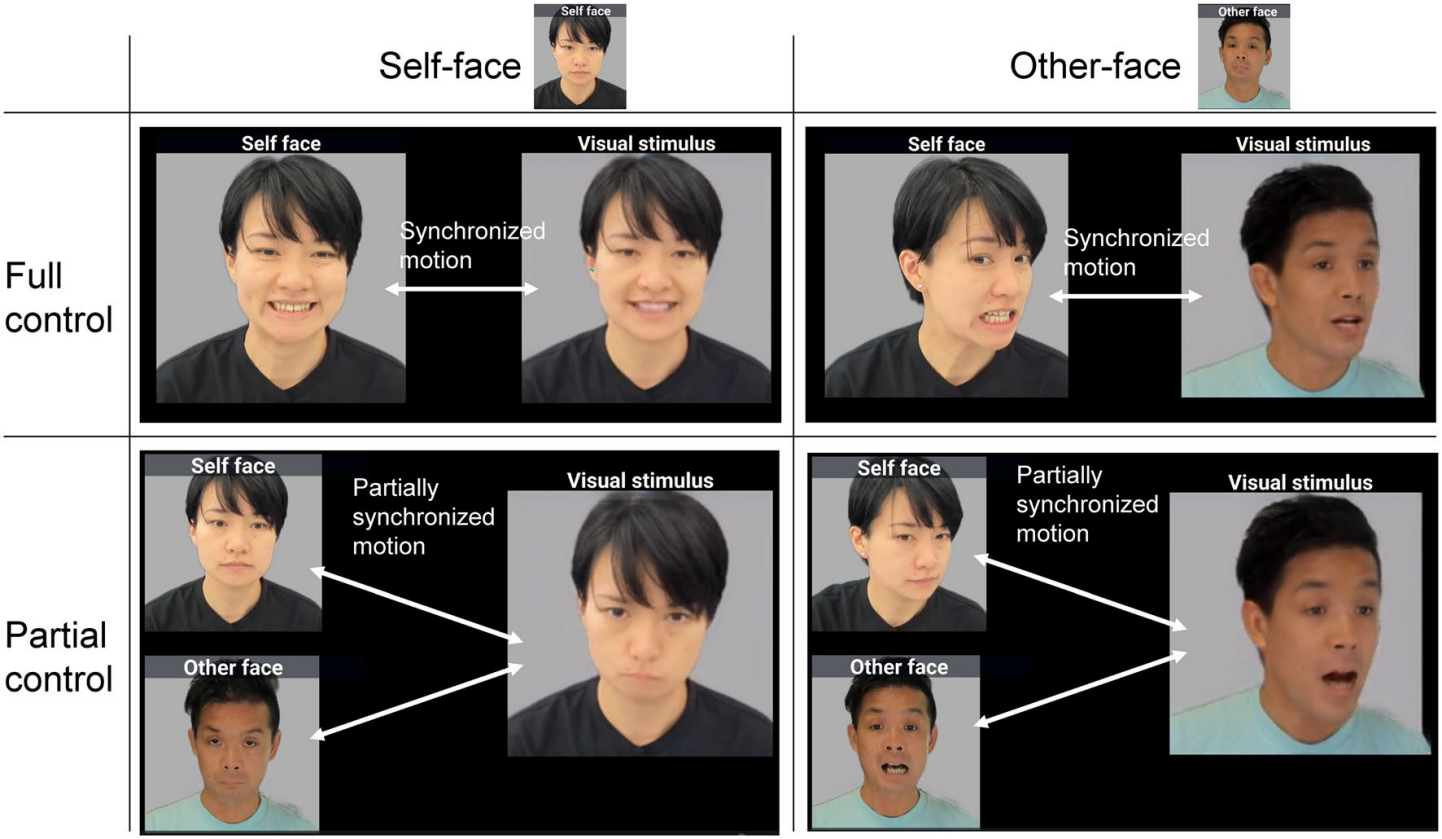
Example of each experimental condition. In the full control condition, the motion of the visual stimulus (i.e., face video) was synchronized with participants’ real-time motion. In the partial control condition, the motion of the visual stimulus was a combination of the participant’s and the experimenter’s real-time motion, thus being partially synchronized with both. Individuals depicted in this figure have provided their informed consent for the use of their images.

The experiment used a 2 × 2 (face × control) within-participant design. First, the factor of face refers to the facial structure information used to generate the face video. It were static photos taken prior to the experiment, and was either participants’ faces (i.e., self-face) or the experimenter’s face (i.e., other-face). Second, the factor of control refers to the extent of how well participants can control the face video. It contains two conditions, full control and partial control. In the full control condition, the motion for the face video was derived from either the participants’ webcam footage or the experimenter’s webcam footage. In the partial control condition, the motion was extracted from both the participants’ and the experimenter’s webcam footage and was averaged before generating the face video that was presented to the participants. Specifically, the LIA abstracted movement vectors from the webcam footage and combined these vectors with a static face photo to generate each frame of the face video. In the partial control condition, the movement vectors from the participant’s webcam and the experimenter’s webcam were mixed in a 50/50 ratio. Therefore, if both people moved to the same extent, their control over the generated face video would be approximately equal. If one person moved while the other remained static, the moving person would have full control over the face video. The actual control of each person was analyzed by calculating the difference between the webcam footage and the presented face video (see S1 File). Note that in all conditions, the face videos were generated by the algorithm. Therefore, even in the self-face & full control condition, the stimuli were not a direct replay of the video captured by the webcam. As a result, all conditions share the same video latency, quality, and other related characteristics. Example videos of the actual face motion and the displayed face video in each condition can be found in the data repository (see Data availability). In summary, there were four experimental conditions: Self-face & full control, other-face & full control, self-face & partial control, and other-face & partial control. Figure 6 shows an example of each condition.

Experiments were conducted individually in a quiet, well-lit room. Participants sat comfortably in front of a monitor and webcam. They were first introduced to the task, and then their photos were taken using the webcam. Thereafter, they completed four practice trials, one for each condition, in a random order. Participants were instructed not to move forward or backward to prevent distortions in the face video. After the practice trials, they proceeded with 20 actual trials, which comprised five repetitions of each condition, presented in a randomized order. During each trial of the partial control condition, the experimenter made head and facial muscle movements in front of a separate webcam. The real-time motions of both the experimenter and the participant were merged to produce the face video. The entire experiment lasted approximately 40 min for each participant.

### Data analyses

To analyze head and facial muscle movements, we first converted the 30 fps recorded videos of participants’ face, experimenter’s face, and the displayed face on the screen into 3D mesh data containing 3D coordinates (x, y, z) of 468 facial landmarks per face, enabling real-time face landmark detection and tracking. To quantify the level of control and characterize actions under different conditions and characterize actions under different conditions, we calculated the magnitude of overall, head, periocular, perioral movements in each condition (see S1 File for the details of action analyses). Furthermore, to investigate how diverse the action plans underlying control detection are, we defined and calculated a diversity index based on the peak switching frequency among overall motion, head movements, and periocular and perioral facial muscle movements (S1 File).

For statistical analyses, we conducted 2 × 2 (face × control) repeated-measures ANOVAs on agency rating, self-identification rating, and the above metrics of head and facial muscle movements to examine the effects of self-face on both the subjective feelings of control and the exploration behaviors underlying it. For significant interactions, we used t-tests to compare the difference between self-face and other-face in each control condition. The significance level was set to 0.025 (= 0.05/2) for multiple comparisons according to Bonferroni correction. All statistical tests were two-sided.

### Supplementary Information

Supplementary Information 1.

Supplementary Information 2.

## Data Availability

The datasets analyzed in this study are available in the Open Science Framework repository, https://osf.io/yf58z/.

## References

[1] Gallagher S (2000). Philosophical conceptions of the self: Implications for cognitive science. Trends Cogn. Sci..

[2] Blakemore S-J, Wolpert DM, Frith CD (1998). Central cancellation of self-produced tickle sensation. Nat. Neurosci..

[3] Blakemore S-J, Frith CD, Wolpert DM (1999). Spatio-temporal prediction modulates the perception of self-produced stimuli. J. Cogn. Neurosci..

[4] Blakemore S-J, Wolpert DM, Frith CD (2002). Abnormalities in the awareness of action. Trends Cogn. Sci..

[5] Frith CD, Blakemore S-J, Wolpert DM (2000). Abnormalities in the awareness and control of action. Philos. Trans. R. Soc. Lond. Ser. B Biol. Sci..

[6] Wen W, Haggard P (2018). Control changes the way we look at the world. J. Cogn. Neurosci..

[7] Karsh N, Eitam B (2015). I control therefore I do: Judgments of agency influence action selection. Cognition.

[8] Wang Q (2012). Infants in control: Rapid anticipation of action outcomes in a gaze-contingent paradigm. PLoS ONE.

[9] Rovee CK, Rovee DT (1969). Conjugate reinforcement of infant exploratory behavior. J. Exp. Child Psychol..

[10] Rochat P, Striano T (1999). Emerging self-exploration by 2-month-old infants. Dev. Sci..

[11] Miyazaki M, Takahashi H, Rolf M, Okada H, Omori T (2014). The image-scratch paradigm: A new paradigm for evaluating infants’ motivated gaze control. Sci. Rep..

[12] Tong F, Nakayama K (1999). Robust representations for faces: Evidence from visual search. J. Exp. Psychol. Hum. Percept. Perform..

[13] Keenan JP (1999). Left hand advantage in a self-face recognition task. Neuropsychologia.

[14] Ma Y, Han S (2010). Why we respond faster to the self than to others? An implicit positive association theory of self-advantage during implicit face recognition. J. Exp. Psychol. Hum. Percept. Perform..

[15] Tacikowski P, Nowicka A (2010). Allocation of attention to self-name and self-face: An ERP study. Biol. Psychol..

[16] Ohata R, Asai T, Imaizumi S, Imamizu H (2022). I hear my voice; therefore I spoke: The sense of agency over speech is enhanced by hearing one’s own voice. Psychol. Sci..

[17] Wen W, Chang AY-C, Imamizu H (2024). The sensitivity and criterion of sense of agency. Trends Cogn. Sci..

[18] Wen W (2020). The active sensing of control difference. iScience.

[19] Wen W (2020). Categorical perception of control. eNeuro.

[20] Wen W, Haggard P (2020). Prediction error and regularity detection underlie two dissociable mechanisms for computing the sense of agency. Cognition.

[21] Wen W, Charles L, Haggard P (2023). Metacognition and sense of agency. Cognition.

[22] Chang, A. Y., Oi, H., Maeda, T. & Wen, W. The sense of agency from active causal inference. *bioRxiv*. 10.1101/2024.01.29.577723 (2024)

[23] Wang, Y., Yang, D., Bremond, F. & Dantcheva, A. Latent image animator: Learning to animate images via latent space navigation. In *International Conference on Learning Representations hal-03714584* (2022).

[24] Sforza A, Bufalari I, Haggard P, Aglioti SM (2010). My face in yours: Visuo-tactile facial stimulation influences sense of identity. Soc. Neurosci..

[25] Wen W, Okon Y, Yamashita A, Asama H (2022). The over-estimation of distance for self-voice versus other-voice. Sci. Rep..

[26] Mori M, MacDorman KF, Kageki N (2012). The uncanny valley. IEEE Robot. Autom. Mag..

[27] Jones KS, Schmidlin EA (2011). Human-robot interaction: Toward usable personal service robots. Rev. Hum. Factors Ergon..

[28] Eitam B, Kennedy PM, Higgins ET (2013). Motivation from control. Exp. Brain Res..

[29] Karsh N (2020). The differential impact of a response’s effectiveness and its monetary value on response-selection. Sci. Rep..

[30] Berlyne DE (1966). Curiosity and exploration. Science.

[31] Modirshanechi A, Kondrakiewicz K, Gerstner W, Haesler S (2023). Curiosity-driven exploration: Foundations in neuroscience and computational modeling. Trends Neurosci..

[32] Golubickis M, Macrae CN (2023). Self-prioritization reconsidered: Scrutinizing three claims. Perspect. Psychol. Sci..

[33] Golubickis M, Macrae CN (2021). Judging me and you: Task design modulates self-prioritization. Acta Psychol. (Amst)..

[34] Wen W, Imamizu H (2022). The sense of agency in perception, behaviour and human–machine interactions. Nat. Rev. Psychol..

[35] Green D, Swets J (1966). Signal detection theory and psychophysics.

[36] Wen W (2021). Perception and control: Individual difference in the sense of agency is associated with learnability in sensorimotor adaptation. Sci. Rep..

