# Supplementary material for: Control over self and others’ face: exploitation and exploration
Source: Sci Rep. 2024 Jul 5;14:15473. doi: 10.1038/s41598-024-66316-2 (PMC11226441; doi:10.1038/s41598-024-66316-2)
Supplement: Supplementary file 1 — Supplementary Information 1. [file 41598_2024_66316_MOESM1_ESM.docx]

**Supplementary Information**

**S1 Fig. Moving errors of the self-face and other-face in each condition**. This index quantifies the discrepancies between the actual webcam footage and the presented stimuli. Three-dimensional mesh data, comprising 3D coordinates (x, y, z) for 468 facial landmarks per face, was extracted from the recorded videos of participants’ movements and the presented stimuli. The average difference in coordinates between the actual facial movement videos and the presented stimuli was calculated for each condition. The value of the coordinates is contingent upon the resolution of the video and, therefore, does not contain actual metric information. A 2 × 2 repeated measures ANOVA revealed a significant main effect of control (*F*(1, 19) = 110.313, *p* < .001, partial η² = .853) and a significant interaction between control and face (*F*(1, 19) = 12.358, *p* = .002, partial η² = .394). The main effect of face was nonsignificant (*F*(1, 19) = 0.334, *p* = .570, partial η² = .017). Post-hoc comparisons showed the motion error was significantly larger in the full control condition when participants controlled someone else’s face than when controlling their own face (*t*(19) = 3.056, *p* = .006, Cohen’s d = 0.681). No significant difference in motion errors between the self-face and other-face conditions was observed in the partial control condition (*t*(19) = 1.428, *p* = .170, Cohen’s d = 0.321). In summary, the motion error results showed that participants had weaker control in the partial control condition compared to the full control condition. Participants also likely exhibited weaker control over someone else’s face than their own. However, this could also be attributed to variations in the abstraction of facial landmark coordinates for different faces.


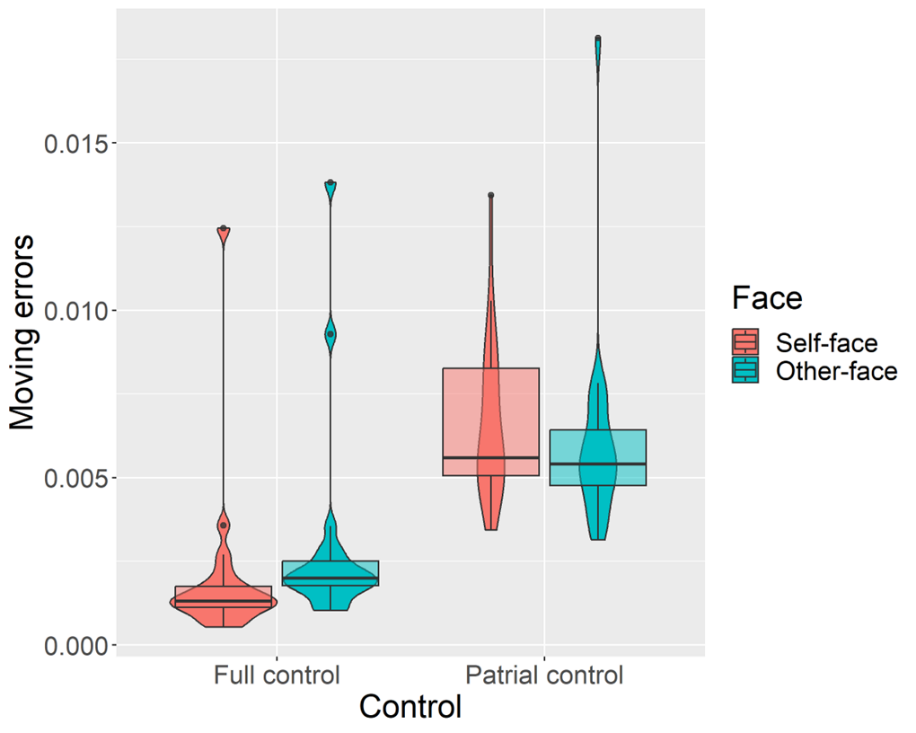


**S2 File. Detailed description of the method used to calculate movement metrics.**
