# Supplementary material for: Control over self and others’ face: exploitation and exploration
Source: Sci Rep. 2024 Jul 5;14:15473. doi: 10.1038/s41598-024-66316-2 (PMC11226441; doi:10.1038/s41598-024-66316-2)
Supplement: Supplementary file 2 — Supplementary Information 2. [file 41598_2024_66316_MOESM2_ESM.docx]

**S2 File. Detailed description of the method used to calculate movement metrics.**

All facial landmarks are indexed in accordance with the MediaPipe FaceMesh Keypoints system (see the figure below; for a full description of the system’s indices, see link^[[1]](#footnote-1)^). For example, the lower left eyebrow area always corresponds to [265, 353, 276, 283, 282, 295] after indexing. The frequency of face-mesh data acquisition corresponded to the frame rate of the video. Movements over time were determined frame-by-frame: For any facial landmark F_1, its movement between frame n (t = T_n) and frame (n+1) (t = T_(n+1)) was determined by the distance between coordinates (x_t_n, y_t_n, z_t_n) and (x_t_n+1, y_t_n+1, z_t_n+1).

To quantify the level of control and characterize actions under different conditions and characterize actions under different conditions, we use the following metrics:

1. *Motion error*, an indicator of the actual level of control, defined as the average difference between actual and displayed face movements.
2. *Overall movements*. We calculated the average movement of all 468 facial landmarks in each trial to measure the overall movements of the whole face, as a result of both head movements and facial muscle movements.
3. *Head movements*. To approximate head movements, we calculated the average movement of coordinates marking the upper face boundary (upper silhouette), given the minimal involvement of the upper face boundary in facial muscle movements. The corresponding MediaPipe FaceMesh Keypoints indices are [10, 338, 297, 332, 284, 251, 389, 356, 127, 162, 21, 54, 103, 67, 109]^[[2]](#footnote-2)^.
4. *Facial muscle movements*. Facial muscle movements were determined by subtracting head movements (as in 3.) from total movements (as in 2.). We then analyze movements of local facial muscle groups, including the periocular and perioral muscles, representing eye and lip movements respectively. To further reduce the confounding effects of head movements, for each facial muscle group, we chose a set of reference points which were minimally affected by local muscle movements. We then subtract movements of these reference points from movements of all coordinates within these facial regions to determine the amount of movements by local muscles only.
   1. For the left periocular region (left eye), the reference points were [362, 263]. Coordinates within this region were [466, 388, 387, 386, 385, 384, 398, 467, 260, 259, 257, 258, 286, 414, 342, 445, 444, 443, 442, 441, 413, 263, 249, 390, 373, 374, 380, 381, 382, 362, 359, 255, 339, 254, 253, 252, 256, 341, 463, 446, 261, 448, 449, 450, 451, 452, 453, 464, 372, 340, 346, 347, 348, 349, 350, 357, 465, 383, 300, 293, 334, 296, 336, 285, 417, 265, 353, 276, 283, 282, 295]^5^.
   2. For the right periocular region (right eye), the reference points were [133, 33]. Coordinates within this region were [246, 161, 160, 159, 158, 157, 173, 247, 30, 29, 27, 28, 56, 190, 113, 225, 224, 223, 222, 221, 189, 33, 7, 163, 144, 145, 153, 154, 155, 133, 130, 25, 110, 24, 23, 22, 26, 112, 243, 226, 31, 228, 229, 230, 231, 232, 233, 244, 143, 111, 117, 118, 119, 120, 121, 128, 245, 156, 70, 63, 105, 66, 107, 55, 193, 35, 124, 46, 53, 52, 65] ^5^.
   3. For the perioral region, the reference points were [48, 278]. Coordinates within this region were [61, 185, 40, 39, 37, 0, 267, 269, 270, 409, 291, 146, 91, 181, 84, 17, 314, 405, 321, 375, 291, 78, 191, 80, 81, 82, 13, 312, 311, 310, 415, 308, 78, 95, 88, 178, 87, 14, 317, 402, 318, 324, 308] ^5^.
5. *Motion diversity*. To investigate how diverse the action plans underlying control detection are, we defined and calculated a diversity index based on the peak switching frequency among overall motion (as in 2), head movements (as in 3), and periocular and perioral facial muscle movements (as in 4). To do this, we computed the moving distance for each type of movement in every frame and normalized these distances within each trial. A sliding window of 10 frames was then used to smooth the data for each trial. Finally, we calculated the number of changes among different types of motion for each trial and averaged them within each condition. This diversity index reflects the frequency with which individuals switch between different movements to test their control over the presented face.


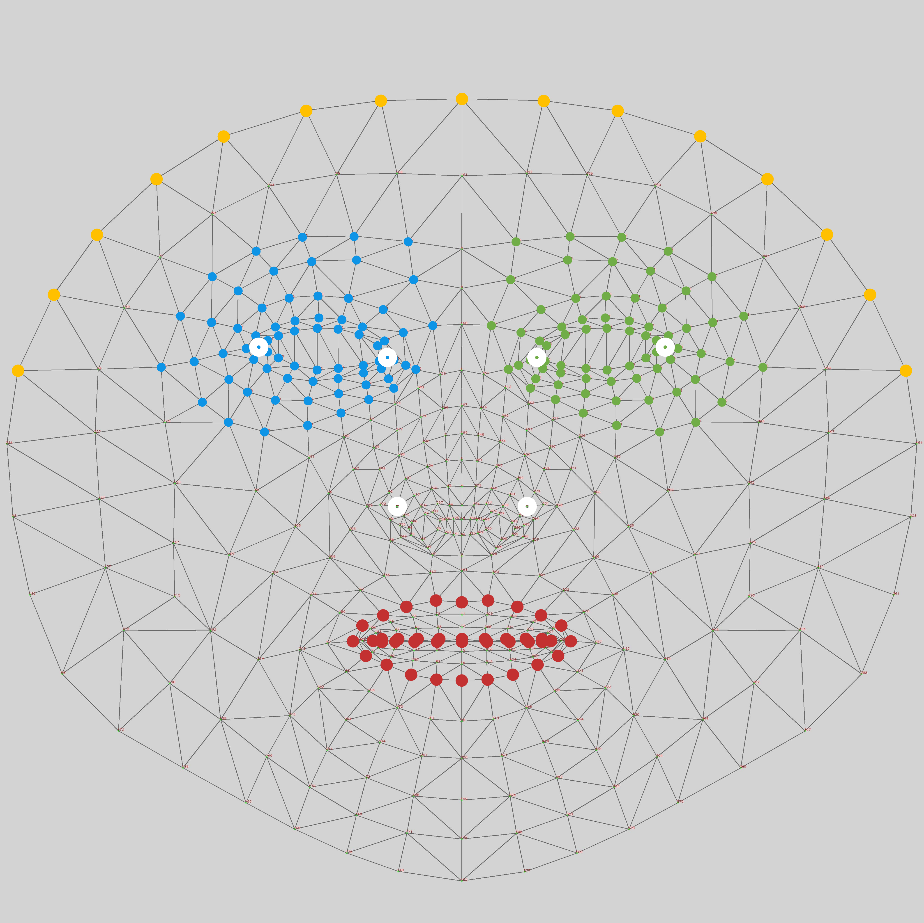


**Facial landmarks used in the action analysis.** The coordinates marked with yellow, blue, green, and red color were used for the calculation of the head movements, the left periocular movements (left eye), the right periocular movements (right eye), and the perioral movements, respectively. The white circled coordinates were used as the reference points for each corresponding region.

1. https://github.com/tensorflow/tfjs-models/tree/master/face-landmarks-detection#mediapipe-facemesh-keypoints [↑](#footnote-ref-1)
2. https://github.com/finalprojectpd117/pythonproject/blob/91ecf27e0a4237c0310d7e7982ef6ce04687d781/final_project/facemesh/views.py [↑](#footnote-ref-2)
